# Cross-validation of prediction equations for estimating body composition in ballet dancers

**DOI:** 10.1371/journal.pone.0219045

**Published:** 2019-07-02

**Authors:** Leilane Lilian Araújo Leal, Giovanna Stefanne Lópes Barbosa, Rannapaula Lawrynhuk Urbano Ferreira, Erikarla Baracho Avelino, Adriana Nunes Bezerra, Sancha Helena de Lima Vale, Bruna Leal Lima Maciel

**Affiliations:** 1 Nutrition Department, Federal University of Rio Grande do Norte, Natal, Rio Grande do Norte, Brazil; 2 Postgraduate Nutrition Program, Federal University of Rio Grande do Norte, Natal, Rio Grande do Norte, Brazil; 3 Endocrinology Department, Onofre Lopes University Hospital, Federal University of Rio Grande do Norte, Natal, Rio Grande do Norte, Brazil; Kennesaw State University, UNITED STATES

## Abstract

**Introduction:**

In sports related to low body weight, such as classical ballet, the assessment of body composition is important for monitoring performance and health status. This study aimed to cross-validate anthropometry and bioelectrical impedance (BIA) predictive equations for estimating body composition of non-professional classical ballet dancers, using dual-energy-X-ray absorptiometry (DXA) as a reference method.

**Materials and methods:**

Thirty-seven female non-professional classical dancers (median age of 19 years), at intermediate/advanced level, were evaluated in a cross-sectional study. Body composition was evaluated by DXA, anthropometry and tetrapolar BIA. Twenty different predictive equations of anthropometry (n = 8) and BIA (n = 12) were used to estimate Body Fat (BF) and Fat-Free Mass (FFM), testing their validity against DXA using the Bland-Altman statistics.

**Results:**

For BF estimated by anthropometry equations, just one equation showed agreement with DXA (r = 0.852, p < 0.0005; p = 0.600 for one sample T-test). According to the Bland-Altman analysis, this equation also showed validity, with the absence of proportional bias. Regarding the predictive BIA equations tested, none were valid for our study group.

**Conclusion:**

Only one of the anthropometric equations, the one proposed by Durnin and Womerley (1974), but none of the BIA equations analyzed, was valid for the evaluation of body composition of the studied classical dancers. Our results emphasize the importance of previous cross-validation of existing equations or the development of specific equations for body composition assessment in specific populations.

## Introduction

Classical ballet is a sport that performing requires good physical training, muscular strength, and endurance [1–4]. The practice of ballet also requires, at different levels of training, a light body with a low percentage of body fat (BF) [5,6] that is placed as necessary for and related to the quality of the dance movements [7–9].

Few girls who practice classical ballet become professionals as adults. Nevertheless, studies have shown that even practitioners of this activity at a non-professional level suffer from pressures for an aesthetic body shape suitable for dancing, which brings impacts on body composition and self-steem [8,10,11]. Thus, studying body composition in ballet dancers is important not only for a better performance, but for the promotion of a healthy nutritional status. Monitoring body composition in dancers can also restrict unnecessary weight loss and the side effects resulting from nutritional, metabolic, and musculoskeletal disorders, such as amenorrhea, eating disorders, and osteoporosis [5,12–14].

In the last decades, dual-energy-X-ray absorptiometry (DXA) analysis has gained credibility and acceptance for body composition assessment, being considered a reference method [15]. Though not commonly available to athletes, DXA has been used to evaluate body composition for different sport modalities, such as basketball, judo, handball, football [16–19] and dance [20–22].

Methods more commonly available to all types of athletes include anthropometry and bioelectrical impedance (BIA) prediction equations, which are used to estimate fat mass (FM) and fat-free mass (FFM) [23,24]. Athletes vary greatly in physique depending on their sport and this is pertinent given that precision errors should be specific to the population studied [23,25]. Additionally, using different prediction equations in populations not similar to that of the development of the equation may give incorrect estimates. For classical ballet dancers, the existing prediction equations were developed using professional dancers of specific ethnicities, one for Greek [26] and the other for American dancers [8]. Thus, the direct application of these equations may give inaccurate results if applied to other populations.

The aim of the present study was to cross-validate BF and FFM anthropometry and BIA prediction equations for body composition assessment, using DXA as a reference method, in non-professional classical ballet dancers. We hypothesized that not all existing anthropometry and BIA prediction equations would be applicable to the study population, considering DXA as a reference method.

## Materials and methods

### Ethics, design and study population

According to the Helsinki Declaration, all participants received detailed information about the study and were invited to give written consent, which was given in the presence of their parents or guardians when necessary. The study was approved by the Ethics Committee of the Onofre Lopes University Hospital, of the Federal University of Rio Grande do Norte (CAAE protocol 38086214.2.0000.5292, acceptance number 925.040).

In the initial screening of the female classical ballet dancers population in Natal-RN/Brazil, in February 2016, we found 50 non-professional classical ballet dancers training in intermediate/advanced level (considered as minimum training of 6 hours per week, using point shoes for at least one year) [27]. These dancers were included in the study from June 2016 to April 2018 according to their availability and acceptance of participating in data collection. Participants answered a questionnaire about their personal data, including self-referred sexual maturation [28], use of medications, and physical activity practice. None of the participants presented any disease or medication use on a regular basis that could interfere with the results, such as antidepressants, antipsychotics, anticonvulsant and hypoglycemic drugs, besides not practicing any other physical activity that exceeded the practice of classical ballet.

### Body composition assessment

Body composition measurements were performed at the Onofre Lopes University Hospital (*HUOL-UFRN*). All measurements were performed on the same morning for each dancer, after 8–10 hours of overnight fasting. Participants were asked not to perform physical exercises or drink alcohol in the 12 hours before the exams. As specific preparation for BIA, participants were asked to ingest at least 2 L of water in the previous day, besides not ingesting coffee and avoiding the use of diuretics (including teas) within 12 hours prior to the examination. To control body water retention, data collection was not performed during the menstrual period of the evaluated subjects. For DXA, as well as BIA, participants were required to remove accessories with metals.

DXA was performed using Lunar DPX L/GE X-ray system (Madison, WI, USA) with the addition of a pediatric software. The examination was performed with the participant lying immobile in dorsal decubitus for evaluation of the entire body, with knees and ankles immobilized with a soft velcro tape. The evaluation determined BF and FFM in percentiles/scores and in pounds, which were converted to kilograms (Kg).

To evaluate the accuracy of DXA scans, the DXA equipment was calibrated daily using a soft tissue phantom, according to the manufacturer’s instructions, allowing a coefficient of variation of ± 3%. The DXA equipment was also routinely evaluated for precision after every 100 patients scan. The Least Significant Change (LSC) was calculated, using the precision calculation tool from the International Society for Clinical Densitometry (ISCD), as recommended (https://www.iscd.org/resources/calculators/precision-calculator/). The LSC calculation was done using repeated measurements in 15 patients scanned three times. Only one technologist performed the assessments and precision assessments. Additionally, assessments were performed two times for dancers from our sample. In these dancers, coefficients of variation (CV) were calculated for FFM and FM and they varied from 0.21% to 1.21%, with mean 0.80%.

For the anthropometric evaluation, an electronic P200C anthropometric scale (*Líder*) was used, with a capacity of 200 Kg and a reported accuracy of ± 100 g for weight measurements and accuracy of ± 0.1 mm for height measurements. Body Mass Index (BMI) was calculated and classified as proposed by the World Health Organization for adults [29]. The z-scores of BMI-for-age and Height-for-age indicators were calculated with the Anthroplus program for adolescents [30].

To measure the skinfolds, a Lange adipometer (Beta Technology Inc., Houston, Texas) was used, with an acuracy of ± 0.1 mm, at the following cutaneous sites: subscapular, tricipital, bicipital, medial axillary, suprailiac, abdominal, thigh and calf. The procedure used for the measurement of the skinfolds followed the recommendations and anatomical sites considered in the literature [31]. Measurements were performed alternately on the participant’s right side and in triplicate. The value used for the calculations was the mean of the triplicates. The maximum difference accepted in the procedures for a new measurement was ± 10% of the value of each measure. Two trained evaluators were responsible for the measurement of skinfolds. Technical error of measurement (TEM) was assessed and intra-evaluator TEM was within ± 5.0% and inter-evaluator TEM within ± 6.0% [32].

For BIA evaluation, the Quantum II tetrapolar tool (RJL Systems, Michigan, USA) was used, following the method described by Lukaski et al. [33]. Resistance (Ω) and reactance (Ω) were measured with the subject lying supine, with four surface self-adhesive spot electrodes and a standard conduction current of 800 PA and 50 kHz. Two electrodes were placed on the dorsal surface of the right hand, and two electrodes were placed on the dorsal surface of the right foot as recommended.

Age, sex and anthropometry data (weight, height, skinfolds) and BIA (resistance and reactance) were used in predictive equations to estimate body composition. The predictive equations of BF and FFM were selected by research in PubMed, Scielo and *Portal Periódicos Capes*, a virtual library available in Brazil, using the following keywords and Boolean operators: body composition AND (predictive OR estimate) AND equation AND validation AND bioimpedance OR anthropometry OR skinfolds). We excluded studies with equations involving different age groups of the present study, equations validated only for males and those developed for populations of specific athletes different from the studied population or for populations with specific diseases. Only studies conducted with similar equipment were used, resulting in the selection of eight predictive anthropometry equations and twelve predictive BIA equations. Of these, three were developed for dancers, as shown in Table 1.

**Table 1:** Characteristics of the selected equations for prediction of Body Fat (BF) and Fat-Free Mass (FFM).

**ANTHROPOMETRY**						
**Reference**	**Subjects**	**Age range**	**Validation method**	**Predictive equation for women**	**R**	**SEE**
**Durnin and Womersley, 1974** [34]	n = 481	16–68 years	HW	BD = (1.1567–0.0717)Log10(TR+BC+SE+SI)	-	0.0116^b^
**Jackson and Pollock, 1980** [35]	n = 249	18–55 years	HW	BD = 1.0994921–0.0009929(TR+TH+SI)+0.0000023(TR+TH+SI)2–0.0001392A	0.842	3.9^c^
**Guedes, 1985** [36]	n = 206	17–27 years	HW	BD = 1.1665–0.0706Log10 (TH + SI + SE)	0.853	0.0053^b^
**Petroski and Pires-Neto, 1995** [37]	n = 281	18–51 years	HW	BD = 1.19547130–0.07513507Log10 (AX + SI + TH + CA)– 0.00041072A	0.829	0.0071^b^
**Sloan, 1962** [38]	n = 50	17–25 years	HW	BD = 1.0764–0.00081 (SI)– 0.00088 (TR)	0.71	0.0082^b^
**Hergenroeder, et al., 1993** [8]	Ballet dancers, n = 112	11–25 years	TOBEC	FFM (kg) = (0.73W)+3.0	0.88^a^	1.5
**Jackson and Pollock, 1975** [39]	Athletes, n = 83	18 a 29 years	HW	BD = 1.096095–0.0006952(TR + SI + TH + AB) + 0.0000011 (TR + SI + TH medial + AB)^2^–0.0000714A	0.85	0.0084^b^
**Slaughter et al., 1988** [40]	n = 310	7–18 years	HW	BF (%) = 1.33(∑DOC)– 0.013(∑DOC)^2^–2.5TR + SE	0.80^a^	-
**BIOELECTRICAL IMPEDANCE**						
**Reference**	**Subjects**	**Age range**	**Validation method**	**Predictive equation for women**	**R**	**SEE**
**Manufacturer Equation–RJL Systems**						
**Chumlea, et al., 2002** [41]	n = 15.903	12–80 years	Dilution of isotopes and multicompartmental models	FFM (Kg) = -9.529 + 0.168W + 0.696H^2^/R + 0.016R	0.83	2.9
**Segal, et al., 1988** [42]	n = 1567	17–62 years	DXA	FFM (Kg) = 5.091+0.6483(H^2^/R) + 0. 1699W	0.800	3.18
**Gray, et al., 1989** [43]	n = 87	19–74 years	HW	FFM (Kg) = 0.00151H^2^–0.0344R + 0.140W − 0.158A + 20.387	0.92	-
**Lukaski, et al., 1986** [33]	n = 114	19–50 years	HW	FFM (Kg) = 0.756(H^2^/R) + 0.110W + 0.107Reac—5.463	0.99^a^	2.3^c^
**Deurenberg, et al., 1991** [44]	n = 827	7–15 years	HW	FFM (Kg) = 0.406[10^4^(H^2^/R)] + 0.360W + 5.58H + 0.56sex– 6.48	0.38^a^	4.4^c^
	n = 827	16–83 years	HW	FFM (Kg) = 0.340[10^4^(H^2^/R)] + 15.34W + 0.273W − 0.127A + 4.56sex– 12.44	0.79^a^	4.1^c^
**Houtkooper, et al., 1992** [45]	n = 94	10–19 years	HW and deuterium dilution	FFM (Kg) = 0.61(H^2^/R) + (0.25W)+ 1.31	0.95	2.1
**Kyle, et al., 2001** [46]	n = 343	20–94 years	DXA	FFM (Kg) = -4.104 + [0.518(H^2^/R)] + (0.231W) + (0.130Reac) + (4.229sex)	0.986	1.72
**Sun, et al., 2003** [47]	n = 1613	12–94 years	HW and DXA	FFM (Kg) = −9.53 + [0.69(H^2^/ R)] + (0,17W) + (0.02R)	0.83^a^	2.9
**Yannakoulia, et al., 2000** [26]	Ballet dancers, n = 42	18–26 years	DXA	FFM (Kg) = 0.247W + 0.214(H^2^/R) + 0.191H − 14.96	0.83^a^	1.45
	Ballet dancers, n = 42	18–26 years	DXA	FFM (Kg) = 0.391W + 0.168H − 0.253TR + 0.144(H^2^/R)– 9.49	0.87^a^	1.32

Subtitles: BF = Body Fat; FFM = Fat-Free Mass; HW = Hydrostatic weighing; TOBEC = Total body electrical conductivity; DXA = dual-energy-X-ray absorptiometry; BD = body density; TR = triceps skinfold; BC = biceps skinfold; SE = subscapular skinfold; SI = suprailiac skinfold; TH = skinfold of the thigh; AX = mean axillary skinfold; CA = medial calf skin fold; AB = abdominal skinfold; R = resistance in ohms; Reac = reactance in ohms; W = weight in kg; H = height in meters; A = age in years. Prediction equation proposed by Hergenroeder et al. [8] for FFM, was considered the BF calculated by the difference between the total weight (kg) of the participant and the result of the equation;

^a^ R^2^, coefficient of determination;

^b^ value in g/ml;

^c^ value in percentage.

Body fat value (Kg) was obtained by subtracting the FFM value from the total mass value. Equations were applied in the present study population according to the corresponding age group: For each equation, we assessed only participants within the same age range originally proposed by the equation. The Siri equation [48] was used to calculate the BF (%) from body density.

### Statistical analysis

The Shapiro-Wilk test and visual inspection of histograms were used to verify the normality of the variables in the study. The variables with normal distributions were presented in table and figures as mean (SD), while the other variables were presented as median (Q1-Q3). All variables were tested for outliers, evaluating box-plots.

Correlation between BF or FFM results generated by the prediction equations and DXA was performed using Pearson’s correlation (r). This analysis was done once the results generated by the equations presented normal distributions.

To give more robustness to the analysis, the one-sample t-test was used to verify if the mean differences between the results of the equations and the results of DXA were significantly different from zero. This test is commonly used for one measured variable and a theoretical expectation of what the mean should be under the null hypothesis. In this analysis, a significant p-value indicated that the tested predictive equation did not present a good agreement with DXA.

Equations with correlation (r with p < 0.05) and agreement (one-sample t-test with p > 0.05) with DXA, were also assessed by the Bland-Altman analysis for cross-validation. The Bland-Altman plots were constructed by placing on the x-axis the mean between the results of the equation and DXA and on the y-axis the difference between the result of the equation and DXA [49]. A central trend line representing the mean of the differences between the equation and DXA was added, and the lines of the minimum and maximum limits were the standard deviations multiplied by ± 1.96. Then, a simple linear regression analysis was performed. This was used to test the presence of proportional bias between the tested equations and DXA, considering the data present in the Bland-Altman plot. The differences between the equation and DXA values were considered as the dependent variable and the mean between the equation and DXA the independent variable. The presence of proportional bias was assumed when a significant p-value (< 0.05) was found, and the equation was not considered valid with DXA as a reference method. For the simple linear regression, the adjusted R^2^, the beta coefficient, which determines whether the bias is positive or negative, the standard error of estimate (SEE) and the p-value were reported.

Statistical analyses were performed with the Statistical Package for Social Sciences version 22.0 (SPSS Inc. Chicago, IL) and the Graph Pad Prism version 3.0 (Graph Pad Software, San Diego, CA) programs.

## Results

In the present study, 38 classical dancers from the initial 50 elegible were evaluated, considering the availability to participate in the study. One dancer was excluded from the sample because of self-referenced prepubescent sexual maturation, which was not compatible with the other participants, totalizing 37 dancers. The median age was 19 (16–24) years, with a range of 14 and 49 years old, which included 17 adolescents and 20 adults who practiced ballet for 10 (5–15) years, with a median of 9 (6–18) hours a week. The results of anthropometry, BIA and DXA are in Table 2. Adult dancers had a BMI considered within the eutrophy range, as well as the adolescents, who presented z-scores with age-appropriate limits. Mean BF was of 28.37 (7.01)% and median FFM of 68.50 (61.69–72.89)%, using DXA. When evaluated separately, the adolescent dancers’ BF of 28.15 (6.55)% was similar to the adults value of 28.61 (7.70)%. None of the studied variables presented extreme values (more than 3 box-lengths from the edge of the box).

**Table 2:** Anthropometry, bioelectrical impedance (BIA) and dual-energy-X-ray absorptiometry (DXA) results of the studied non-professional classical ballet dancers.

Variable	Mean (SD) or Median (Q1—Q3)
**Anthropometry**	
Body Mass (kg)	51.44 (5.49)
Body Height (m)	1.60 (0.42)
BMI (Kg/m^2^)	20.70 (2.49)
BMI/age (z-score)^a^	-0.45 (-0.59)
Hight/age (z-score) ^a^	-0.60 (0.44)
Skinfolds (mm)	
Subscapular	11.72 (3.42)
Biciptal	9.40 (6.50–10.78)
Triciptal	15.00 (10.90–18.38)
Average axillary	10.92 (4.09)
Suprailiac	15.00 (9.40–23.25)
Abdominal	19.67 (7.09)
Thigh	25.87 (6.30)
Calf	10.92 (4.09)
**BIA**	
Resistance (ohms)	672 (70)
Reactivity (ohms)	68 (64–76)
Total body water (%)^b^	50.76 (4.22)
**DXA**	
Body Fat (%)	28.37 (7.01)
Body Fat (Kg)	13.04 (10.29–17.38)
Fat-Free Mass (%)	68.50 (61.69–72.89)
Fat-Free Mass (Kg)	32.37 (30.41–35.35)

Subtitles: BMI = body mass index;

^a^classification for individuals aged 14 to 19 years (n = 19).

^b^predicted by the manufacturer’s equation.

**Fig 1:**
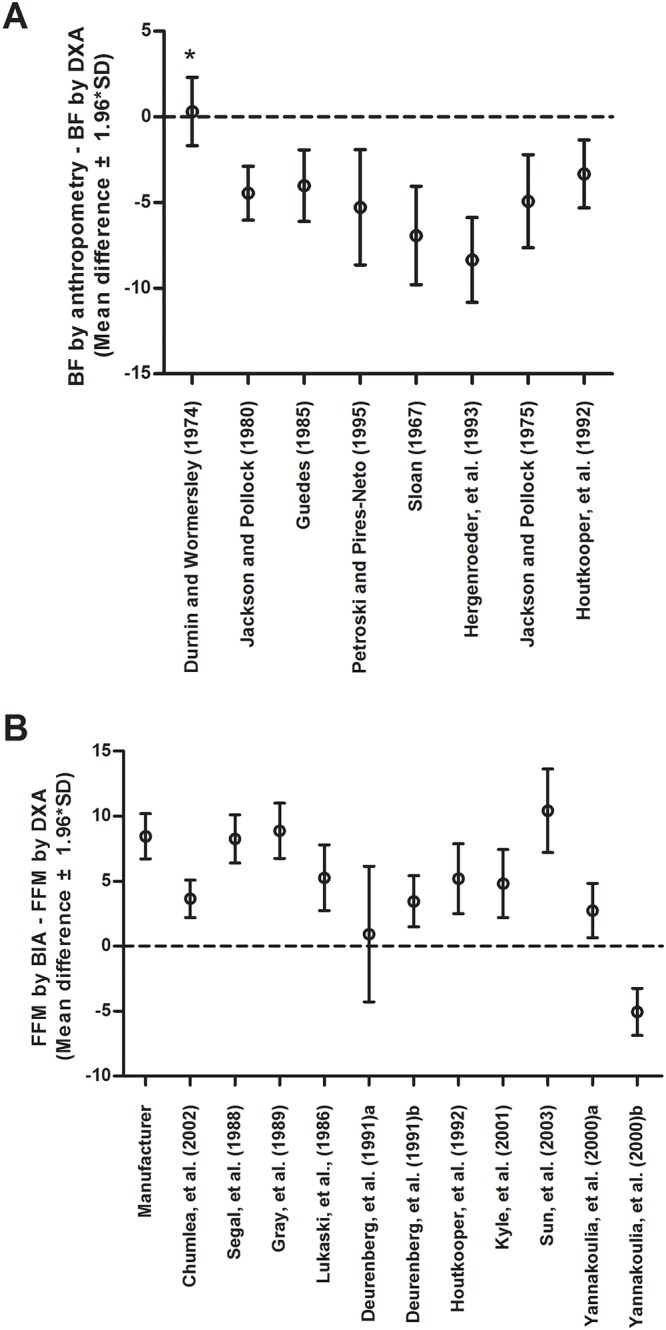
Mean differences between A) Body Fat (BF) from the anthropometry equations and BF given by DXA and B) Fat-Free Mass (FFM) from the BIA equations and FFM given by DXA. Error bars represent ± 1.96 x standard deviations of the mean differences. *Equation presented one-sample t-test with p = 0.600, no significant difference for results obtained from BF by the equation against DXA. The other predictive equations presented p values < 0.05 for the one-sample t-test. The doted line represents the reference value expected in the one-sample t-test (zero).

Among the anthropometry equations for BF (%) prediction, only the equation proposed by Durnin and Womersley (1974) [34] showed a significant correlation with DXA (r = 0.852, p <0.0005) (Table 3) and no significant difference in the BF (%) estimate when compared to DXA (one-sample t-test, p = 0.600) (Fig 1A). All other equations tended to underestimate BF (%) when compared to DXA, generating negative differences (Fig 1A). In the case of the twelve BIA equations for FFM (%) prediction, some presented correlation with DXA (Table 4), but all presented differences that were significantly higher or lower than zero for the results obtained from FFM against DXA (Fig 1B).

**Table 3:** Comparison of Body Fat (BF) measured by different anthropometry equations using the dual-energy-X-ray absorptiometry (DXA) as a reference method.

Predictive equation	n	BF (%) by Equation Mean (SD)	BF (%) by DXA Mean (SD)	Pearson’s correlation analysis	
				r	p value
Durnin and Womersley, 1974 [34]	20	29.18 (4.51)	28.87 (7.39)	0.852	< 0.0005
Jackson and Pollock, 1980 [35]	15	24.07 (5.59)	28.52 (7.46)	0.945	< 0.0005
Guedes, 1985 [36]	17	23.69 (4.29)	27.70 (7.21)	0.868	< 0.0005
Petroski and Pires-Neto, 1995 [37]	12	23.52 (4.03)	28.76 (7.06)	0.838	0.001
Sloan, 1962 (26)	13	23.70 (3.80)	30.63 (7.61)	0.859	< 0.0005
Hergenroeder, et al., 1993 [8]	30	21.15 (0.60)	29.48 (6.76)	0.672	< 0.0005
Jackson and Pollock, 1975 [39]	11	23.71 (5.44)	28.29 (7.40)	0.890	0.001
Slaughter et al., 1988 [40]	15	24.60 (3.25)	27.92 (5.77)	0.830	< 0.0005

**Table 4:** Comparison of Fat-Free Mass (FFM) measured by different Bioelectric impedance (BIA) equations using the dual-energy-X-ray absorptiometry (DXA) as a reference method.

Predictive equation	n	FFM (%) by Equation Mean (SD)	FFM (%) by DXA Mean (SD)	Pearson’s correlation analysis	
				r	p value
Manufacturer Equation–RJL Systems	35	76.24 (2.83)	70.86 (7.67)	0.507	0.002
Chumlea, et al., 2002 [41]	35	71.43 (4.56)	70.86 (7.67)	0.565	< 0.0005
Segal, et al., 1988 [42]	27	75.01 (5.83)	69.73 (7.71)	0.720	< 0.0005
Gray, et al., 1989 [43]	19	75.25 (5.07)	70.04 (8.29)	0.768	< 0.0005
Lukaski, et al., 1986 [33]	19	71.63 (6.85)	70.04 (8.29)	0.708	0.001
Deurenberg, et al., 1991 [44]	07	72.71 (2.63)	74.56 (7.17)	0.377	0.404
	28	70.25 (3.44)	69.93 (7.63)	0.626	< 0.0005
Houtkooper, et al., 1992 [45]	19	73.29 (4.17)	70.27 (7.63)	0.331	0.166
Kyle, et al., 2001 [46]	17	72.05 (5.91)	71.34 (7.72)	0.680	0.003
Sun, et al., 2003 [47]	35	78.22 (8.06)	70.86 (7.67)	-0.057	0.743
Yannakoulia, et al., 2000 [26]	16	69.65 (3.91)	69.83 (6.85)	0.791	< 0.0005
	16	64.29 (5.12)	69.34 (8.92)	0.788	0.003

**Fig 2:**
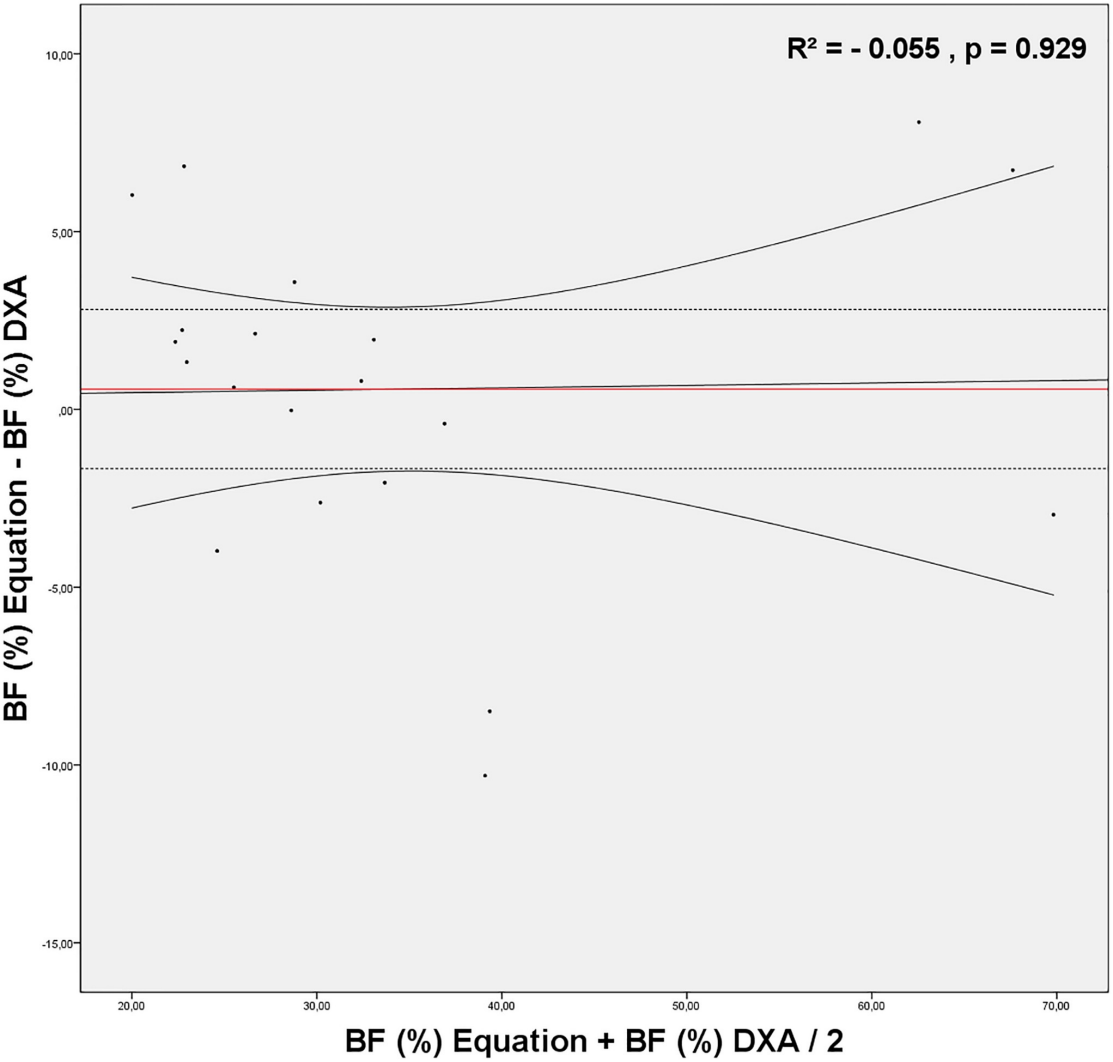
Bland-Altman plot for Body Fat (BF) estimated by the anthropometry equation proposed by Durnin and Womersley (1974) and BF estimation by dual-energy-X-ray absorptiometry (DXA) as a reference method. The doted lines represent 95% limits of agreement (±1.96 SD). The red line represents the regression line between mean BF by the equation and BF by DXA and mean differences between BF equation and BF DXA. Beta coeficient = 0.021 and SEE = 4.91. Curved lines represent the 95% confidence interval for the regression line.

Therefore, for results from the Durnin and Womersley [34] equation, the Bland-Altman plot was constructed and a simple linear regression was performed to verify the cross-validity of the results against those obtained by DXA. The plot revealed that the equation did not underestimate or overestimate BF, with the mean difference of 0.57%. No proportional bias between DXA and the equation was found, once the regression did not explain the difference found in the two methods of BF estimation (R^2^ = -0.055, p = 0.929) (Fig 2).

## Discussion

Classical ballet is a sport with performance intrinsically related to body composition and shape [50]. The present study aimed to evaluate cross-validity of eight predictive equations of anthropometry and twelve BIA equations to estimate BF and FFM in classical ballet dancers. To our knowledge, no studies have performed this type of evaluation, using DXA as a reference method and considering non-professional dancers.

The weekly training volume found in the present study was lower than that found in other studies, showing that our results are compatible with a non-professional intermediate/advanced practicing [21,26,51]. Although the dancers of the study presented a eutrophic BMI, they presented a higher BF% when compared to other studies in professional dancers, with percentages ranging from 17.5% to 24.6% [26,52]. Not only time and level of practice can be considered the causes for the higher BF% observed in our study. Ethnicity and higher age and may also be determining variables for the different results [24,53]. As an example, Eliakim et al. [20] found in Caucasian adolescent ballet dancers a lower BF% than that found in the present study [21].

Few studies have analyzed body composition of Brazilian ballet dancers [54–57], one of which studied pre-professional ballet dancers [54] and none used DXA. These studies reported mean ages of 13.1 to 36.8 years and mean BF% values of 15.2% to 21.0%. Most of the studies used skinfolds measurements with Slaughter’s et al. equation [40], and only one used BIA [56] with Houtkooper’s et al. equation [45]. These studies reinforce that the availability and use of DXA in Brazil is still limited for dancers, and it is necessary to validate more accessible methods to evaluate this population.

The Durnin & Wormersley’s [34] equation was the only applicable to the population in our study, once the final Bland-Altman analysis revealed no significant difference in measurements or proportional bias. This equation was developed from research conducted in England in 1974 with 209 males and 272 females, mostly sedentary, aged between 16 and 72 years old.

Interestingly, our analysis showed that the three equations originally developed with dancers [8,26] were not applicable in the evaluated sample. We did not find other studies cross-validating these three equations. The equation proposed by Hergenroeder et al. [8] was developed using 112 professional ballet dancers in the Houston Ballet Academy, with a mean age of 14.4 (1.3) years, dancing 16 (12) hours per week, and validated with the TOBEC method. The authors concluded that the simplicity of the developed equation, using only weight (Kg), could facilitate studies and clinical practice.

Yannakoulia et al. [26] developed two equations validated with 42 dancers of a professional ballet school in Athens. One of the equations was considered the best because it needed only BIA data in its formula. The second equation needed BIA and anthropometry data, which was recognized by the authors more challenging to apply. The equation was also validated with DXA, the ballet dancers had a mean age similar (21 years) to that found in our study (19 years) and were not considered professionals. Dancers trained more hours per week (28.3 hours) than those in the present study, and this may justify the non-applicability of the equations of Yannakoulia et al. [26] in the population herein analyzed.

Studies have reported a correlation between BF and FFM estimates using anthropometry and BIA predictive equations with DXA in several populations, including dancers [21,26,58]. However, no study has sought to cross-validate the existing anthropometry or BIA predictive equations for dancers using DXA or any other method as a reference method.

One limitation of our study is the difference in the standardization for the skinfolds measurements in the different equations. To decrease bias and enable data collection, we obtained anthropometric data using a standardized protocol [31], compatible with most of the selected equations. In addition, most of the mathematical models found in the literature were developed with the hydrostatic weighing method as a reference, as well as methods such as total body electrical conductivity and isotope dilution. These methods differ from DXA because they have different principles of body composition, and this may be a limitation. Our small sample size, determined by the fact we used a very homogeneous specific population of athletes, might also be a limitation to further generalization.

Of importance, there is a tendency in clinical practice to individually follow up some skinfolds to assess the evolution of body composition [23]. This follow up is a problem, especially in evaluating people practicing sports related to aesthetics, since it makes a body analysis impossible, and may induce unnecessary anxiety and fixation about particular sites of the body, inherent to the shape of an individual. Embracing this kind of assessment also limits the quality of collective planning measures and consensuses for actions to improve body composition of populations.

On the other hand, if an anthropometry or BIA predictive equation are going to be used in clinical care to estimate body composition, professionals should be aware to carefully observe the equations, since criteria such as gender, ethnicity, age, and type of physical activity may be important variables that influence the results. Our study showed that not all anthropometry and BIA prediction equations tested would apply to the study population, accepting our initial hypothesis. Thus, previous cross-validation of predictive equations from the literature for a specific population, as we have done in the present study, or developing new equations for specific populations, such as classical ballet practitioners, are of relevance once it can improve the assessment of that population.

## Conclusion

The results of the present study showed that from the twenty body composition predictive equations tested, only the Durnin and Womersley [34] equation was applicable to the estimation of body composition in the studied classical ballet dancers. Our data reinforce the importance of cross-validating existing predictive equations to estimate body composition of specific populations or developing equations, considering the characteristics of the population of interest.
